# Comparative proteomic profile of *Aspergillus niger* in response to polytetrafluoroethylene and irradiated polytetrafluoroethylene for enhanced bioremoval

**DOI:** 10.1007/s10532-025-10215-4

**Published:** 2025-12-04

**Authors:** Khaled Saeed, Shaimaa Abdelmohsen Ibrahim, Ola M. Gomaa

**Affiliations:** Radiation Microbiology Department, National Center for Radiation Research and Technology (NCRRT), Egyptian Atomic Energy Authority (EAEA), Cairo, Egypt

**Keywords:** *Aspergillus niger*, Bioremoval, Proteomic profile, Irradiation, Electron beam, Polytetrafluoroethylene (PTFE)

## Abstract

Polytetrafluoroethylene (PTFE) is a widely used fluoropolymer known for its chemical stability and resistance to degradation, making it a persistent environmental pollutant. The bioremediation of PTFE has proven challenging due to its inert nature. The aim of the present study is to characterize how changes in PTFE and irradiated PTFE may affect the proteomic profile of *Aspergillus niger* to propose protein biomarkers and depict bioremoval strategies. The results show that irradiating PTFE causes structural and spectral changes that increase with the increase of electron beam irradiation doses at 20, 40, 80, 160, and 320 kGy as compared to the control. PTFE and irradiated PTFE were added to a 24 h *Aspergillus niger* culture, and the proteomic profile was studied using quantitative protein assay and a high-throughput Ultra Performance Liquid Chromatography (UPLC) proteomics approach. The resultant chromatograms show that peak shifts can serve as a rapid indicator of PTFE and irradiated PTFE, highlighting the potential of proteomic profiling as a rapid screening tool. Energy Dispersive X-Ray (EDX) mapping images show fluoride attached to *A. niger* mycelia, while quanitative SPADNS Fluoride assay revealed deflourination % of 28.0 and 31.6% for 80 and 320 kGy irradiated PTFE culture, respectively, as compared 11.2% for non-irradiated PTFE. These findings suggest that 1) high electron beam irradiation doses enhance PTFE degradation, 2) the proteomic profile can be used as a biomarker to detect the presence of PTFE or irradiated PTFE, and 3) *A. niger* can be further exploited for both PTFE and irradiated PTFE bioremoval via deflourination or adsorption on mycelial network. Further research is needed to enhance the deflourination process.

**Supplementary Information:**

The online version contains supplementary material available at 10.1007/s10532-025-10215-4.

## Introduction

Per and poly-fluorinated substances (PFAS) have been used since the 1940s. They are anthropogenic compounds that consist of thousands of individual species, each with different applications, but they share the same hydrophobic nature and chemical and mechanical stability (Gluge et al 2020). All PFAS consist of a carbon-fluorine bond (C-F), which requires high energy of 536 kJ/mole to break them (Son et al 2020); therefore, they are called “forever compounds” and listed as Persistent Organic Pollutants (POPS) due to their persistence, toxicity, and bioaccumulative nature in the environment (Jin et al 2021). One of the most commonly used PFAS compounds is Polytetrafluoroethylene (PTFE), commonly known as Teflon. It is widely recognized for its nonstick properties in cookware and its exceptional chemical, thermal, and electrical stability in various applications. Over time, coatings degrade when exposed to high temperatures or when coatings are damaged, leading to the release of micro or nano PTFEs in food or water. (Evich et al 2022). Generally, ingestion of PTFE particles results in oxidative stress, inflammation intracellular signaling pathway alterations (Yu et al 2025). The Environmental Protection Agency (EPA) has set guidelines for maximum detection limits of all per- and poly-flourinated compounds along with a timeframe for industries to comply with set rules and regulations in order to limit the release of those compounds in drinking water by 2027 (https://www.epa.gov/sdwa/and-polyfluoroalkyl-substances-pfas). Research on the removal of per- and poly-fluorinated compounds relies on using membrane separation, such as nanofiltration, reverse osmosis (RO), or adsorption membranes (Jin et al 2021), or using Granular Activated Carbon (GAC) (Burkhardt et al 2022). However, chain length, functional group, and molecular weight determine the extent of removal using GAC and membranes (Burkhardt et al 2022). PFAS compounds can also be removed using coagulation and flocculation (Hubert et al 2024), photocatalysis (Zelekew et al 2024), or surfactant-assisted ultrasonication (Awoyemi et al 2025). Electrochemical advanced oxidation process (EAOP) was reported to be efficient but has a relatively slow reaction rate and high energy consumption (Xu et al 2024). Ultrasonication was reported to affect the structure and size of PTFE (Pu et al 2025). Since the destruction of per- and poly-fluorinated substances requires high energy, irradiation is considered one of the potent tools to break the C-F bond. Electron beam irradiation was reported to induce this breakup; however, depending on the type of compound and chain length, a 90% degradation would require between 500 and 1000 kGy (Londhe et al 2024). While chemical and physical approaches result in acceptable removal levels of PFAS, their disadvantages were reported to include the appearance of toxic by-products or high energy cost; therefore, microbial biodegradation can be introduced as an eco-friendly and low-energy approach. PFAS bioremediation relies on the redox potential of microbial cells within their media to catalyze PFAS breakdown via deflourinating enzymes. The redox potential of microorganisms is dependent on oxygen availability, rendering aerobic conditions more favorable than anaerobic conditions (Niu et al 2024). However, both oxidation and reduction could result in PFAS biodegradation by inserting oxygen atoms or by adding extra electrons to the C-F bond, respectively. While numerous studies have focused on the degradation of PFAS, particularly PFOS and PFOA using single or combined treatments (Sharma et al 2024), other fluorinated polymers such as PTFE remain unexplored due to their chemical inertness and high stability. Biodegradation studies focused on bacterial species such as *Pseudomonas* sp., *Godrondia* sp., *Acidimicrobium* sp., and *Mycobacterium* sp., which can degrade PFAS compounds within 2 to 100 days, depending on initial PFAS concentration. On the other hand, a few fungal strains, such as *Phanerocheate chrysosporium* and *Trametes versicolor* were reported to degrade PFAS compounds within 28 days (Zhang et al 2022). However, fungal response to highly fluorinated Therefore, from this standpoint, the aim of the present study aims 1) to use Electron beam irradiation to modify PTFE to enhance its accessibility to *Aspergillus niger*, and 2) to compare *A. niger* proteomic response to native versus irradiated PTFE as a rapid identification method.

## Materials and methods

### Microorganism and growth media

*Aspergillus niger* was isolated from soil previously and identified using 18S rRNA of ITS region. The ITS sequence was deposited in the National Center for Biotechnology Information (NCBI) Gene Bankit nucleotide sequence database under accession number JF437542 (Gomaa et al 2013). The fungus was grown in Yeast Extract Sucrose (YES) media composed of the following per L: Yeast Extract, 4 g, Sucrose 20 g, KH_2_PO_4_ 1 g, MgSO_4_ 0.5 g, pH 6.2. The media was supplemented with 1 M CaCl_2_. A spore suspension was prepared from a 7-day-old culture plated on YES-Agar. Spores were scraped with sterile 1% Tween 80; spore count was 17 × 10^5^ cfu/mL.

### Polytetrafluoroethylene (PTFE)

PTFE powder (Alfa Aesar, Thermo Fischer, GmbH) was suspended in water at a 1000 µg/10 mL concentration. Dynamic Light Scattering (DLS) and zeta potential were performed using Anton Paar Litesizer DLS 500 (Back scatter, 25 °C, solvent water). UV–visible scan was performed using UV–Visible spectrophotometer (SPECORD 210 plus, analytic Jena) scan 200–700 nm.

### Polytetrafluoroethylene (PTFE) Irradiation

PTFE powder was exposed to Electron Beam radiation at 0, 20, 40, 80, 160 (80 × 2 cycles) and 320 (80 × 4 cycles) kGy using ICT electron beam accelerator (3 MeV) Energy 90 kw, Beam ampere Max up to 30 mA, Max conveyor speed 16 mm/min, Max high voltage (3000kv) at the National Center for Radiation Research and Technology, Cairo, Egypt. Absorbance of PTFE in aqueous media was monitored at absorbance maxima obtained from the abovementioned experiment. Transmission Electron Microscope (TEM) images were obtained for PTFE and irradiated PTFE (at 80 and 320 kGy) using TEM 100CX at 80 kV. The powder samples were placed on carbon-coated grids, and images were obtained at a magnification of 26000X, Scale bar:100 nm, using AMT camera system. Irradiated PTFE samples (at 80 and 320 kGy) were analyzed for functional groups by FTIR-Spectrophotometer (Bruker, Germany) in the spectral range 400 to 4000 cm^−1^. The obtained spectrum was analyzed for the presence of possible functional groups and compared with that obtained from a non-irradiated PTFE sample.

### PTFE bioremoval using *Aspergillus niger*

*Aspergillus niger* spore suspension was used to inoculate 250 mL Erlenmeyer flasks with 100 mL YES working volume. The effect of PTFE concentrations and inoculum size (%) was determined based on *Aspergillus niger* cell dry weight (CDW). PTFE was added in the following concentrations: 0,1, 2, 4, 8 µg/mL, using 5% inoculum size. While inoculum sizes tested were: 0, 2.5, 5, 10, 20% and 4 µg/mL. The flasks were incubated at 30 °C and 150 rpm for 24 h. The flasks were incubated for another 48 h. At the end of the incubation period, all flasks were filtered using sterile Mira cloth, filtered mycelia were washed and dried overnight at 50 °C to calculate CDW.

### Proteomic profile for *A. niger* cultures in the presence of PTFE, irradiated PTFE, and control cultures

The optimal PTFE concentration and inoculum size from the previous experiment were used in the following experiment. A final concentration of 4 µg/mL of PTFE and irradiated PTFE (80 and 320 kGy) was added to the 24 h culture flasks inoculated with 10% *A. niger*. The flasks, along with a control set (*A.niger* without PTFE), were left to incubate for another 48 h. At the end of the incubation period, all flasks were filtered using sterile Mira cloth. The culture filtrates from all flasks were used for protein precipitation. Each culture filtrate was added to 95% ethanol (9:1), incubated for 60 min at −20 °C, then centrifuged at 10,000 rpm for 10 min at 4 °C. The precipitate was washed with PBS pH 7 and centrifuged under the same conditions before being resuspended in PBS. The suspension was passed through a sterile 0.22 µm filter before protein analysis using Ultra Performance Liquid Chromatography (UPLC), Hitachi, Japan. The column used was a short C18, 3.5 µm, 4.6 × 75 mm column, Part No. WAT066224. The mobile phase A: Trifluoracetic acid aq. solution: acetonitrile (90:10), mobile phase B: Trifluoracetic acid aq. solution: acetonitrile (40:60), detection at 280 nm for proteins. Protein profiles were obtained, and area% for the 4 samples were plotted as an overlapping scatter plot using Excel. Protein quantification for PTFE and irradiated PTFE was performed using Biuret protein assay kit according to the manufacturer’s instructions. Absorbance was measured at 550 nm; protein concentration was calculated using the standard protein available in the kit. For statistical analysis, ANOVA and Tukey post-hoc analysis were performed using R software to compare the significance of protein concentrations among the four groups: *A.niger* grown in YES media (Group A), *A. niger* grown in the presence of PTFE (Group B), and *A. niger* grown in the presence of irradiated PTFE at 80 kGy (Group C) and at 320 kGy (Group D).

### Removal of PTFE and irradiated PTFE by *Aspergillus niger* mycelia

The residual fluoride was quantified using SPADNS method. About 10 mL of mixed acid-zirconyl-SPADNS solution was added to 50 mL of the samples, mixed well, and the absorbance of the generated red solution from each sample was measured at 570 nm using a spectrophotometer. Deflourination (%) was calculated according to the following equation:

$$\mathrm{Deflourination\%}=\frac{Cft}{Cox2}\times 100$$

where C_ft_ is fluoride concentration after treatment, C_o_ is theoretical fluoride derived from PTFE mass, and 2 comes from the two F atoms in each -CF_2_-unit (Pu et al 2025). Statistical analysis, ANOVA, and Tukey post-hoc analysis were performed using R software to compare the significance of deflourination (%) among the three groups: non-irradiated PTFE, 80 kGy irradiated PTFE, and 320 kGy irradiated PTFE.

*Aspergillus niger* pellets were collected from each flask (control, PTFE, and irradiated PTFE). The mycelia were left to dry on a clean cover slide and were observed using Scanning Electron Microscopy (SEM). Images were captured using a Zeiss evo15 scanning electron microscope (Germany). The samples were placed on brass stubs using double-sided adhesive tape and coated with a thin layer of gold under reduced pressure. The images were captured at magnifications of 3000X using an electron beam high voltage of 20 kV. Energy Dispersive X-Ray (EDX) mapping was performed using a Zeiss evo15 scanning electron microscope (Germany). Samples were analyzed before and after incubation. The samples were placed on brass stubs using double-sided adhesive tape EDX and the elements C, O, and F were analyzed in control, PTFE, and irradiated PTFE samples.

## Results

### PTFE characterization

**Fig. 1:**
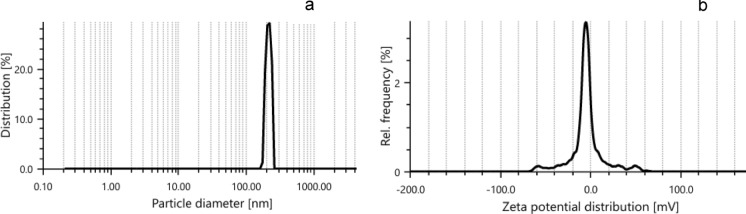
DLS **a** and Zeta potential **b** for PTFE suspended in water, showing size 213 nm and −7.6 mV, respectively

**Fig. 2:**
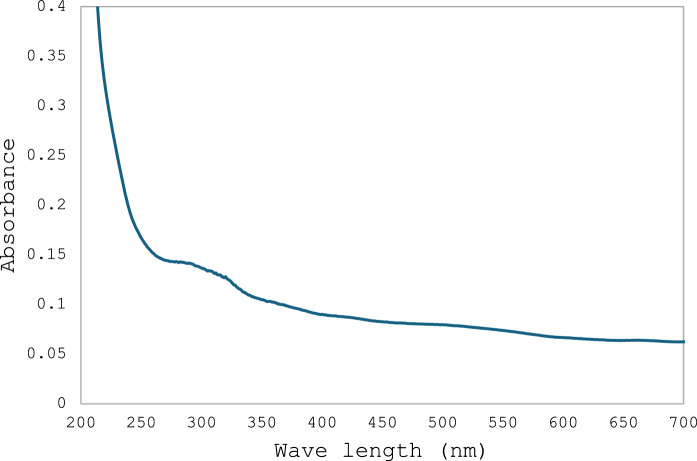
UV–visible spectrum for PTFE

Figure 1 shows that the size is uniform and is about 213 nm, while the overall charge is −7.6 mV as detected using a Zeta analyzer. The UV–Visible spectrum for PTFE shows a single peak at 293 nm when dissolved in water (Fig. 2). This absorbance maxima will be used for following up experiments after irradiating PTFE.

### Electron beam irradiation of PTFE powder

**Fig. 3:**
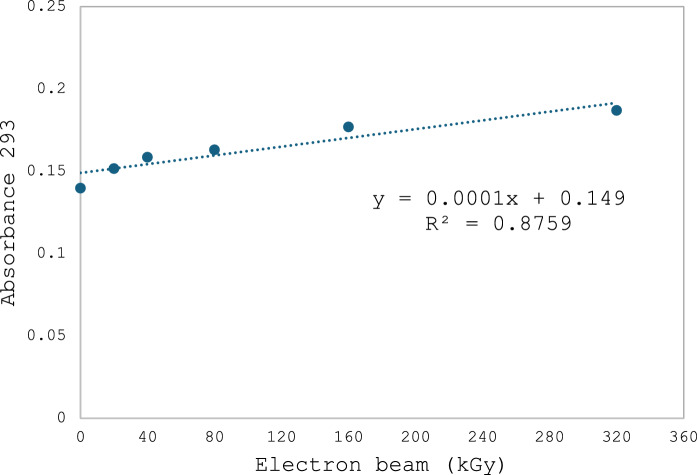
Correlation between UV absorbance at 293 nm and electron beam irradiation doses

**Fig. 4:**
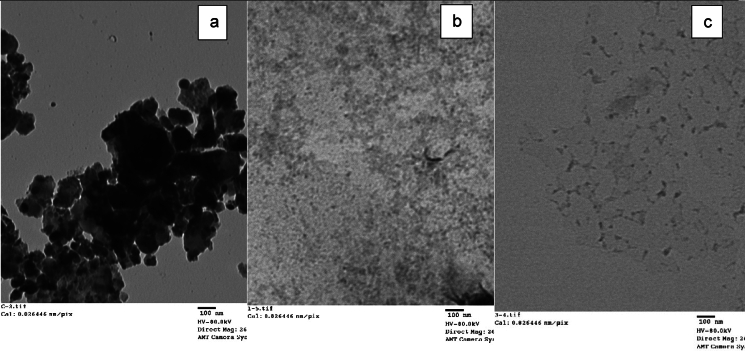
TEM images for PTFE **a**, irradiated PTFE at 80 kGy **b** and 320 kGy **c**. Scale bar: 100 nm and magnification: 26000X

**Fig. 5:**
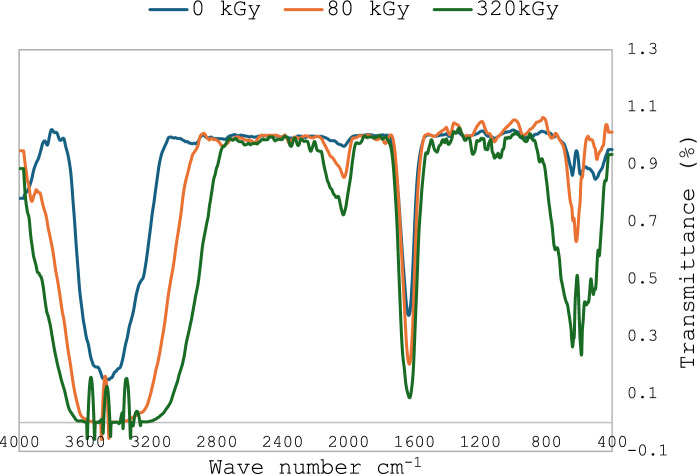
FTIR spectrum for PTFE and irradiated PTFE (80 and 320 kGy)

PTFE was exposed to different radiation doses at 0, 20, 40, 80, 160, and 320 kGy. Figure 3 shows the correlation of absorbance at 293 nm with the irradiation dose. The results show a straight-line correlation with R^2^ of 0.8497, which represents a good correlation coefficient that confirms the increase in absorbance with an increase in irradiation dose. Figure 4 shows that exposing PTFE granules to different doses resulted in changes in morphological shape and size. High density particles in image a, while reduction in PTFE particles were observed upon exposure to 80 and 320 kGy as seen in images b&c, respectively. PTFE is represented as repeating unit: – (CF₂–CF₂)ₙ– therefore, –CF₂– group dominates the IR spectrum. The main peaks characteristic of PTFE are stretching C-F vibration from 1140 to 1200 cm^−1^, CF_2_ showing a peak between 500–640 cm^−1^, and C-F bond represented between 1100 and 1350 cm^−1^ (Fig. 5).

### *A. niger* growth in the presence of PTFE

**Fig. 6:**
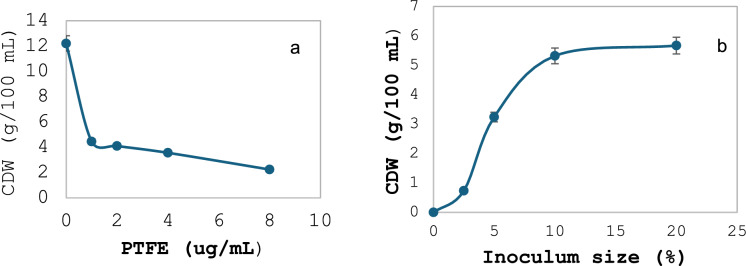
CDW (g/100 mL) *Aspergillus niger* (5% inoculum size) in the presence of different PTFE concentrations (**a**) and with increasing inoculum size (%) and in the presence of 4 µg/mL PTFE (**b**)

The PTFE concentration to be added to *A. niger* spores was determined using two experiments that aimed to determine the maximum PTFE concentration and optimal spore suspension. The results showed that adding increasing concentrations of PTFE to *A. niger* spore suspensions (5% inoculum size) directly resulted in a decrease in *A. niger* growth as represented by the Cell Dry Weight (CDW) from 12.2 to 2.24 g/100 mL culture when the PTFE concentrations were 0 and 8 µg/mL, respectively (Fig. 6a). On the other hand, the CDW increased when the *A. niger* inoculum size increased from 3.24 to 5.67 g/100 mL when the inoculum size increased from 5 to 20%, respectively (Fig. 6b).

### Protein profile and protein content

**Fig. 7:**
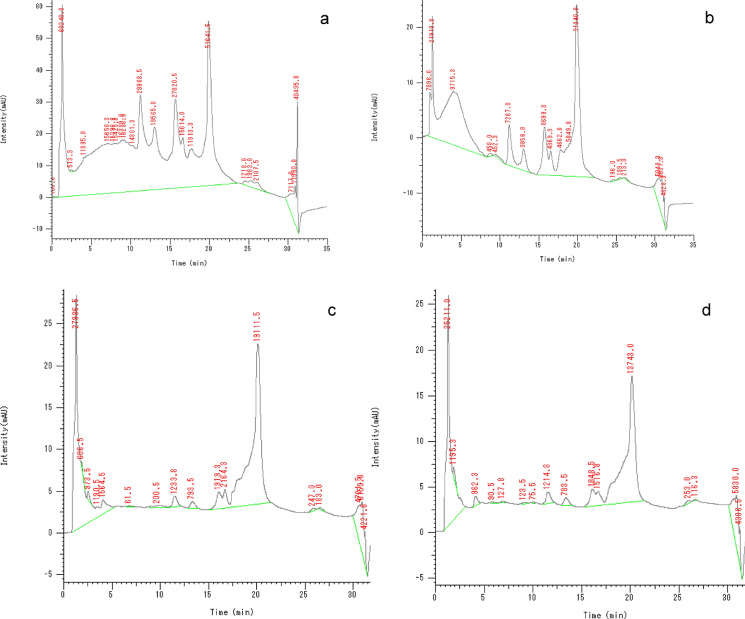
Proteomic profile for *A.niger* grown in YES media **a**, in the presence of PTFE **b**, and in the presence of 80 kGy irradiated PTFE **c** and in the presence of 320 kGy irradiated PTFE **d**

**Fig. 8:**
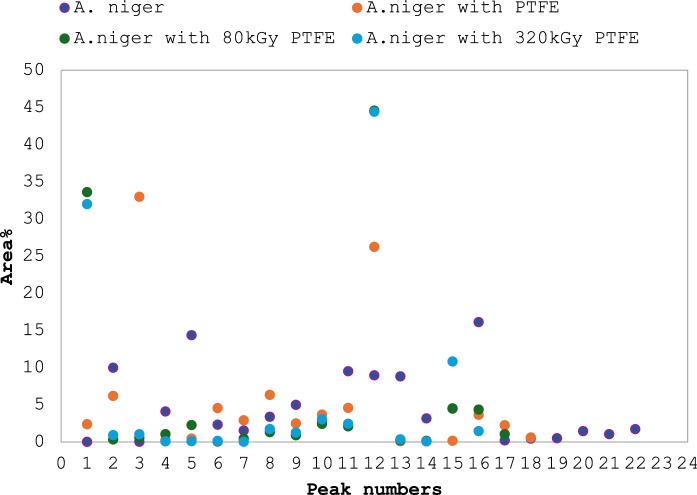
Scattered plot representing proteomic profile peaks (area%) for *A.niger* grown in YES media (purple dots), in the presence of PTFE (orange dots) and in the presence of 80 kGy irradiated PTFE (green dots) and in the presence of 320 kGy irradiated PTFE (blue dots)

**Fig. 9:**
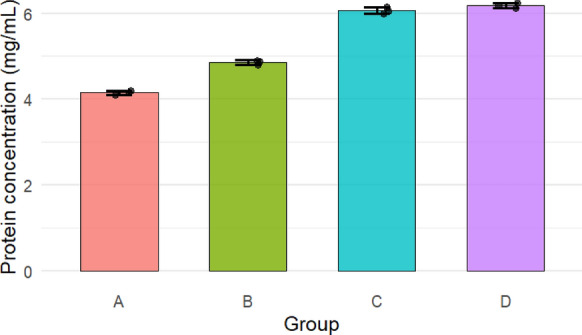
Protein concentration (mg/mL) *A.niger* grown in YES media (group A), *A. niger* grown in the presence of PTFE (group B), and *A. niger* grown in the presence of irradiated PTFE at 80 kGy (group C) and at 320 kGy (group D). P < 0.001

Figure 7a–d shows that adding PTFE and irradiated PTFE to *A.niger* resulted in a shift in the proteomic profile. Figure 8 and Table 1 show that the area % for those peaks increased from 0.036 and 8.9 in control samples to 32.97 and 26.97 in the presence of PTFE. On the other hand, peaks 5 and 16 in control samples decreased from 14.35 and 16.125 to 0.458 and 3.655 in the presence of PTFE, respectively. An evident increase in area % of peaks 1 and 12 can be seen in the presence of irradiated PTFE where they showed an increase from 2.39 to 43.862 and 26.97 to 44.413 for PTFE and irradiated PTFE, respectively. This shows that irradiation induced an increase in peak intensity of 18.77 and 1.65-fold, respectively. Figure 8 also shows that the number of peaks was reduced from 22 to 17 when PTFE was added, and further decrease took place when irradiated PTFE was added. On the other hand, Fig. 9 shows an increase in protein concentrations upon the addition of irradiated PTFE to the *A. niger* culture media. Statistical analysis was performed using Two-way ANOVA, P value > 0.001. Tukey Psot-Hoc shows that there is significant difference between proteins for *A.niger* grown in YES media (group A), *A. niger* grown in the presence of PTFE (group B), and *A. niger* grown in the presence of irradiated PTFE at 80 kGy (group C) and at 320 kGy (group D). However, there is no significant difference between groups C and D where P value was < 0.001 (S1).

**Table 1:** Area % of major peaks for *A.niger*, *A. niger* grown in presence of PTFE and *A. niger* grown in presence of irradiated PTFE (80 and 320 kGy)

Sample	Peak 1	Peak 3	Peak 5	Peak 12	Peak 16
*Aspergillus niger*	0.015	0.036	14.35	8.9	16.125
*Aspergillus niger* + PTFE	2.39	32.97	0.458	26.97	3.655
*Aspergillus niger* + 80 kGy PTFE	38.161	1.327	2.276	44.565	4.36
*Aspergillus niger* + 320 kGy PTFE	43.862	1.674	0.222	44.413	1.46

### Effect of adding PTFE and irradiated PTFE on *A. niger* and its bioremoval efficiency

**Fig. 10:**
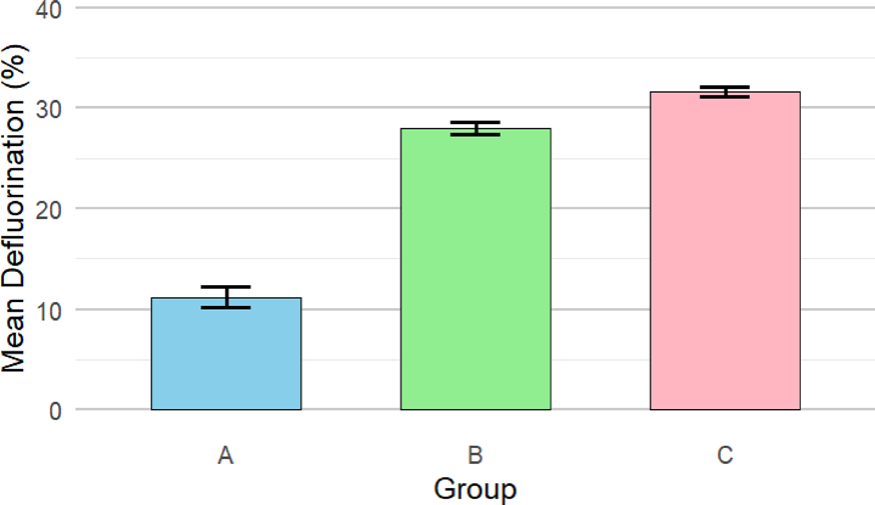
Mean deflourination(%) after incubating PTFE with *A. niger* cultures (Group A), and irradiated PTFE at 80 kGy (Group B) and 320 kGy (Group C). P < 0.001

**Fig. 11:**
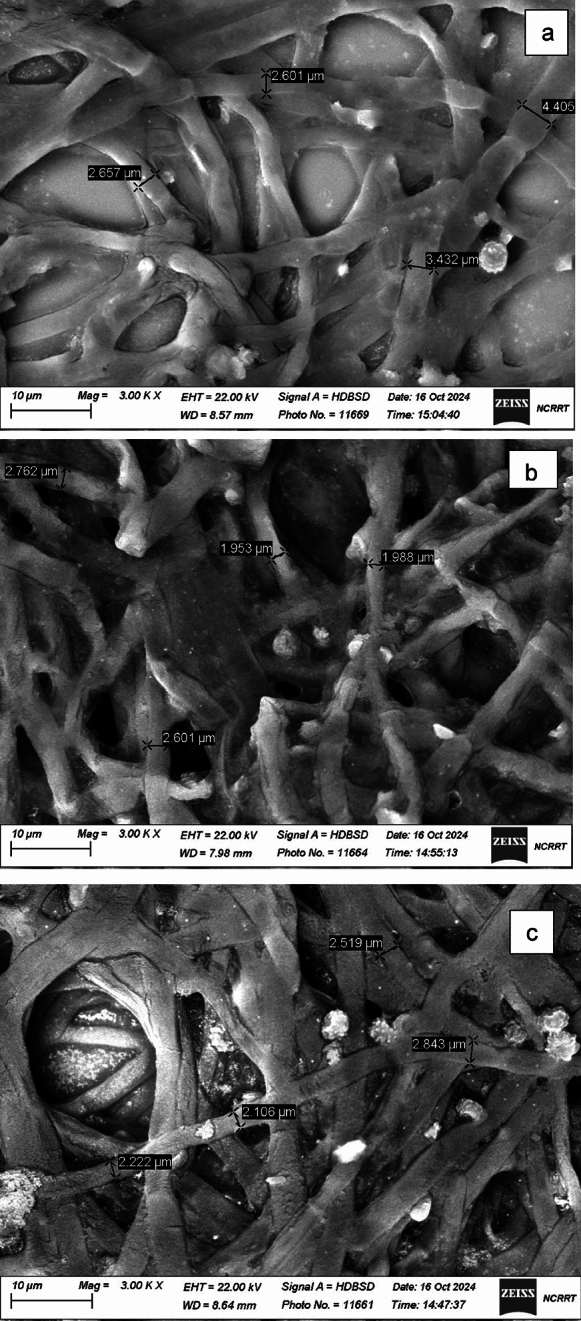
SEM micrographs showing *A. niger* mycelia in the presence of PTFE **b** and irradiated PTFE **c** as compared to control **a**. Scale bar 10 µm and magnifications at 3000X

**Fig. 12:**
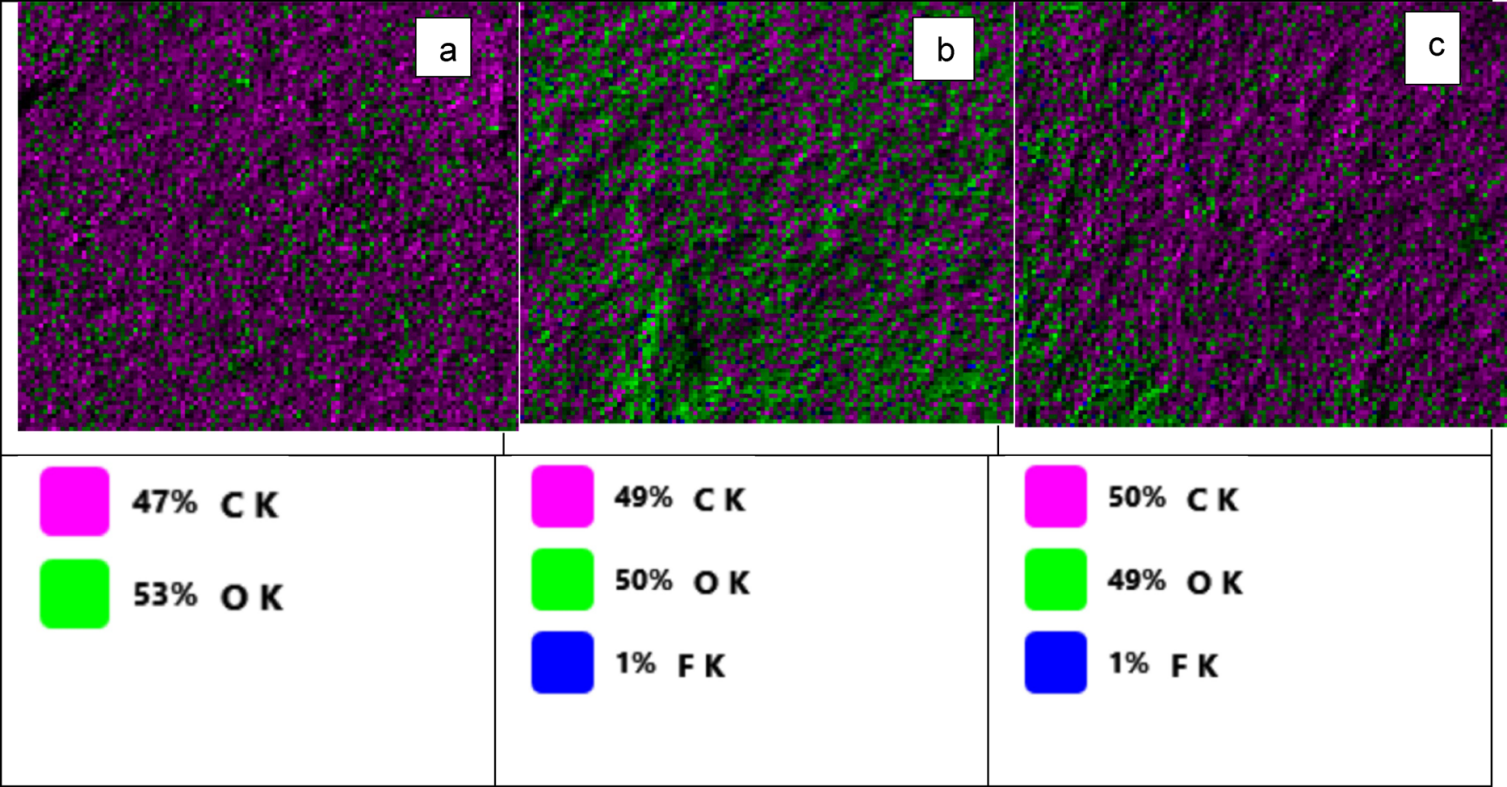
EDX mapping for C, O and F on the surface of *A.niger* grown in YES media **a**, in presence of PTFE **b** and Irradiated PTFE **c**

**Fig. 13:**
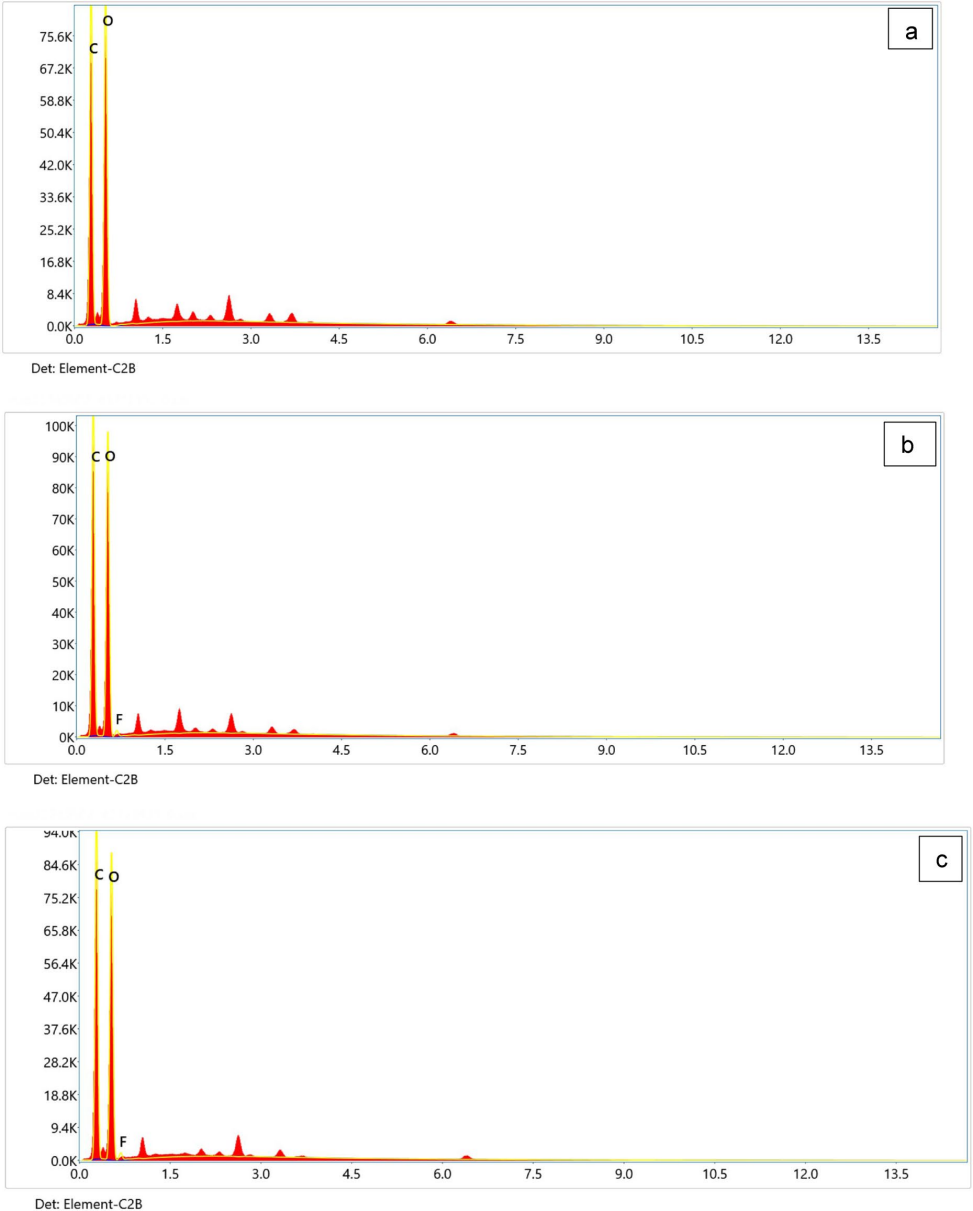
Elemental analysis spectrum for *A. niger* **a***, A.niger* with PTFE **b** and *A. niger* with irradiated PTFE **c**

Figure 10 represents the deflourination % as detected using SPADNS spectrophotometric assay. The results show deflourination of 11.2% for non-irradiated PTFE (group A), 28.0% for 80 kGy irradiated PTFE (group B), and 31.6% for 320 kGy irradiated PTFE (group C) incubated with *A. niger* cultures, respectively. P value was < 0.001, which is highly significant between the three groups. Tukey Post hoc test showed significance between groups A and B, C and A, but not between B and C(S2). To study the effect of growing *A. niger* in PTFE and irradiated PTFE, the fungal mycelia were collected at the end of the cultivation period and examined using SEM and EDX mapping. The results in Fig. 11 show that mycelial width varied in size, where they were in the range between 2.6–4.4, 1.98–2.7, and 2.1–2.8 in *A.niger* control cultures, in the presence of PTFE, and in the presence of irradiated PTFE, respectively. Figure 12 represents elemental mapping of *A. niger* mycelia captured using EDX. The results show that 1% F was adsorbed on the surface of mycelia in the presence of both PTFE and irradiated PTFE, while carbon atom content were 49 and 50%, respectively, as compared to control sample (53%). Figure 13 and S3 represent the EDX spectra and elemental analyis for the same samples and confirm the results in Fig. 12.

## Discussion

The present study focuses on two key aspects: the first was to evaluate PTFE modification using electron beam irradiation to induce biodegradation, and the second was to compare *A. niger* proteomic profile in response to native PTFE and irradiated PTFE as a rapid qualitative detection method. PTFE is a white powder with low solubility in water at high concentrations and is highly soluble in polar solvents such as acetone and chloroform. It is also dispersed in water if added in low concentrations. The particle distribution tested using Dynamic Light Scattering confirmed a homogenous suspension. Overall surface charge showed a slight negative charge, as shown through zeta potential distribution. UV–visible spectrum showed a distinct peak at 293 nm, which is within the 250–500 nm reported range for PTFE. Electron beam irradiation of PTFE showed distinct structural and morphological changes at 320 kGy as seen in TEM images, which show a reduced size of PTFE upon exposure to increased electron beam irradiation. This coincides with Pu et al (2025), who reported that using physical treatment such as ultrasonication has resulted in a reduction of PTFE size. While Yu et al. (2025) research aimed at reducing PTFE weight loss and reported 91% weight loss at 270 °C and exposure at 7.5 MGy (which is 7500 kGy). Londhe et al (2024) suggested doses between 500 and 1000 kGy to break down per- and poly-fluorinated compounds. In the present study, we opted for electron beam irradiation doses of a maximum of 320 kGy, which were enough to induce structural changes, as an initial step before biodegradation. Although microorganisms can utilize synthetic polymers as energy sources, initiating degradation through enzymatic cleavage of polymer chains (Amaral-Zettler 2022), the use of ionizing radiation before bioremediation is considered a promising synergistic approach to accelerate the breakdown of plastics (Afify et al 2025). In the present study, a low concentration of PTFE was added to *Aspergillus niger* cultures to ensure dispersion in water. Although PTFE is inert, chemically resistant, and non-biodegradable, *A. niger* may still interact with it, leading to the upregulation of stress-related proteins and oxidative stress enzymes (Odebode & Adekunle 2019; Yaakoub et al 2022). At the same time, the expression of proteins linked to primary metabolism or growth may decline, indicating a metabolic shift toward survival and stress management rather than active growth (Srikanth et al 2022). This can be seen in the figures representing the decrease in growth with increasing PTFE concentration.

*A. niger* proteomic profile fingerprint in the presence of PTFE and irradiated PTFE was studied for the rapid detection of both native and irradiated PTFE. The proteomic profile of *Aspergillus niger* showed distinct changes in the presence of PTFE that were different from those shown in the presence of irradiated PTFE, reflecting the fungus’s adaptive response. This can be considered a biomarker that can be tracked and used to identify the presence of such molecules using UPLC charts. The peaks appear after 20 min of flow time and offer a rapid method for detection as opposed to separating the nanoparticles from the aqueous media to identify them using conventional spectroscopy techniques. Metabolic signatures were used to assess cellular response to the presence of PTFE nanoparticles and were considered a sensitive method to detect underlying metabolic mechanisms to detect NPs induced toxicity (Xuan et al 2024).

To assess PTFE degradation, cleavage of C-F into F^−^ is calculated as deflourination %. The result of using electron beam irradiation and biodegradation by *A. niger* in the present study led to an increase in deflourination from 11.2 to 30.6% when electron beam irradiation was used as pretreatment. The obtained percentage is close to that obtained by Pu et al (2025) after ultrasonication. Physical treatment of microplastics caused structural and morphological changes that resulted in an increase in microbial adhesion to microplastics and consequently enhanced biodegradation (Afify et al 2025). FTIR spectroscopic analysis confirms that changes in PTFE exposed to electron beam irradiation suggest that initial carbonylation takes place (Senna et al 2001). This results in changing PTFE from hydrophobic to hydrophilic, which facilitates its adsorption onto the fungal mycelial cell wall. This explains that in our work, fluoride was present on *A. niger* mycelia, as shown in EDX mapping images and EDX spectra. The FTIR results show the presence of the -OH group, and the cultivation of *A. niger* under shaking conditions provides O_2_, which leads to stronger evidence that oxidative defluorination took place. However, non-irradiated PTFE released fluoride as well in the media, suggesting that *A. niger* has an enzyme that performs defluorination. Both aerobic and anaerobic deflourination were reported; however, aerobic conditions are more effective due to the higher microbial metabolic rate of aerobic microorganisms, which leads to enhanced PFAS biodegradation (Zhang et al 2022). Oxidoreductases are the main enzyme class that are responsible for PFAS degradation (Amin et al 2025); other reports mentioned that metalloenzymes are also involved in deflourination (Wang and Liu 2020). Previous RNA Seq of *A.niger* under study in YES media showed an increase in the expression of several oxidoreductase genes that were involved in dye biodegradation (Gomaa et al 2017). Since oxidative defluorination is represented as:

C-F + 2e^−^ + 3H^+^ + O_2_ → C–OH + H_2_O + F^−^ (Wickett et al. 2022), therefore, based on the previous results, we suggest that oxidative defluorination in the present study can be the involved pathway for PTFE removal by *A. niger*. Further research is needed to couple electron beam irradiation and oxidative biodegradation to reach higher defluorination results. Suggestions for enhancing biodegradation of per- and poly-fluorinated substances and further breaking C–C and C-F bonds include the use of quorum-sensing signaling molecules, adding carbon-based and metal-based materials to the media (Huang et al 2025).

## Conclusion

This study provides the first insight that sheds light on how fungi differentially respond to native versus irradiated PTFE at the proteomic level, revealing a distinct protein profile triggered by the polymer's altered structural properties. These findings provide the foundation for developing 1) bio-based treatments targeting fluoropolymer pollution that can be coupled with electron beam irradiation as pre-treatment, thus offering a sustainable approach to mitigating the environmental impact of these persistent materials, and 2) the proteomic profile can act as a biomarker that enables rapid identification of the presence of PTFE in its native or irradiated form. The research outcome represents an initial step for identifying possible approaches to harness upscaling of PTFE biodegradation. More research is required to upscale biodegradation and identify the proteins that are secreted in response to PTFE and irradiated PTFE for potential use as a probe in biosensors.

## Supplementary Information

Below is the link to the electronic supplementary material.

Supplementary file1 (DOCX 16 KB)

Supplementary file2 (DOCX 16 KB)

Supplementary file3 (DOCX 17 KB)

## Data Availability

No datasets were generated or analysed during the current study.
