# Supplementary material for: Comparative proteomic profile of Aspergillus niger in response to polytetrafluoroethylene and irradiated polytetrafluoroethylene for enhanced bioremoval
Source: Biodegradation. 2025 Dec 4;37(1):6. doi: 10.1007/s10532-025-10215-4 (PMC12678457; doi:10.1007/s10532-025-10215-4)
Supplement: Supplementary file 1 — Supplementary file1 (DOCX 16 KB) [file 10532_2025_10215_MOESM1_ESM.docx]

S1: ANOVA and Tukey multiple comparison of means for Fig. 8

| > summary(anova_result)  Df Sum Sq Mean Sq F value Pr(>F)  Group 3 8.609 2.8697 839.9 2.47e-10 ***  Residuals 8 0.027 0.0034  ---  Signif. codes: 0 ‘***’ 0.001 ‘**’ 0.01 ‘*’ 0.05 ‘.’ 0.1 ‘ ’ 1 |
| --- |
|  |
| \|  \| \| --- \| |

Tukey multiple comparisons of means

95% family-wise confidence level

Fit: aov(formula = Protein_conc ~ Group, data = data)

$Group

diff lwr upr p adj

B-A 0.7066667 0.55383099 0.8595023 0.0000021

C-A 1.9133333 1.76049766 2.0661690 0.0000000

D-A 2.0266667 1.87383099 2.1795023 0.0000000

C-B 1.2066667 1.05383099 1.3595023 0.0000000

D-B 1.3200000 1.16716432 1.4728357 0.0000000

D-C 0.1133333 -0.03950234 0.2661690 0.1598790
