# Supplementary material for: Comparative proteomic profile of Aspergillus niger in response to polytetrafluoroethylene and irradiated polytetrafluoroethylene for enhanced bioremoval
Source: Biodegradation. 2025 Dec 4;37(1):6. doi: 10.1007/s10532-025-10215-4 (PMC12678457; doi:10.1007/s10532-025-10215-4)
Supplement: Supplementary file 2 — Supplementary file2 (DOCX 16 KB) [file 10532_2025_10215_MOESM2_ESM.docx]

**S2: ANOVA and Tukey multiple comparison of mean for Fig.9**

anova_model <- aov(Defluorination ~ Group, data = data)

> summary(anova_model)

Df Sum Sq Mean Sq F value Pr(>F)

Group 2 713.0 356.5 207.9 2.88e-06 ***

Residuals 6 10.3 1.7

---

Signif. codes: 0 ‘***’ 0.001 ‘**’ 0.01 ‘*’ 0.05 ‘.’ 0.1 ‘ ’ 1

|  |  |  |  |  |  |  |  |
| --- | --- | --- | --- | --- | --- | --- | --- |

| > TukeyHSD(anova_model) | | |  |
| --- | --- | --- | --- |
| Tukey multiple comparisons of means | | | |
| 95% family-wise confidence level | | | |
|  |  |  |  |
| Fit: aov(formula = Defluorination ~ Group, data = data) | | | |
|  |  |  |  |
| $Group |  |  |  |
| diff lwr upr p adj | | | |
| B-A 16.800000 13.5197248 20.080275 0.0000100 | | | |
| C-A 20.433333 17.1530581 23.713609 0.0000030 | | | |
| C-B 3.633333 0.3530581 6.913609 0.0335016 |  |  |  |
