# Supplementary material for: Comparative proteomic profile of Aspergillus niger in response to polytetrafluoroethylene and irradiated polytetrafluoroethylene for enhanced bioremoval
Source: Biodegradation. 2025 Dec 4;37(1):6. doi: 10.1007/s10532-025-10215-4 (PMC12678457; doi:10.1007/s10532-025-10215-4)
Supplement: Supplementary file 3 — Supplementary file3 (DOCX 17 KB) [file 10532_2025_10215_MOESM3_ESM.docx]

**S3: Elemental analysis tables for Fig. 12**

**Table 1a: Elemental analysis table for *A. niger.***

| Element | Weight % | Atomic % | Net Int. | Error % | R* | A** | F*** |
| --- | --- | --- | --- | --- | --- | --- | --- |
| C K | 45.62 | 52.78 | 1722.64 | 9.71 | 0.9340 | 0.1249 | 1.0000 |
| O K | 54.38 | 47.22 | 1878.57 | 10.08 | 0.9419 | 0.0895 | 1.0000 |

*R represents the correlation coefficient, **A represents the absorption effect, and *** F represents the fluorescence effect.

**Table 1b: Elemental analysis table for *A.niger* with PTFE.**

| Element | Weight % | Atomic % | Net Int. | Error % | R* | A** | F*** |
| --- | --- | --- | --- | --- | --- | --- | --- |
| C K | 46.73 | 54.00 | 1948.26 | 9.70 | 0.9341 | 0.1247 | 1.0000 |
| O K | 51.79 | 44.92 | 1932.57 | 10.09 | 0.9420 | 0.0875 | 1.0000 |
| F K | 1.48 | 1.08 | 40.78 | 13.68 | 0.9452 | 0.0581 | 1.0000 |

*R represents the correlation coefficient, **A represents the absorption effect, and *** F represents the fluorescence effect.

**Table 1c: Elemental analysis table for *A. niger* with irradiated PTFE.**

| Element | Weight % | Atomic % | Net Int. | Error % | R* | A** | F*** |
| --- | --- | --- | --- | --- | --- | --- | --- |
| C K | 46.92 | 54.20 | 1989.69 | 9.71 | 0.9341 | 0.1247 | 1.0000 |
| O K | 51.36 | 44.54 | 1940.86 | 10.11 | 0.9420 | 0.0871 | 1.0000 |
| F K | 1.72 | 1.26 | 48.47 | 13.35 | 0.9452 | 0.0584 | 1.0000 |

*R represents the correlation coefficient, **A represents the absorption effect, and *** F represents the fluorescence effect.
